# First report on the complete mitochondrial genome of the deep-water scalpellid barnacle *Arcoscalpellum epeeum* (Cirripedia, Thoracica, Scalpellidae)

**DOI:** 10.1080/23802359.2018.1532844

**Published:** 2018-10-26

**Authors:** Se-Joo Kim, Hyun Mi Kang, Laure Corbari, Benny K. K. Chan

**Affiliations:** a Genome Editing Research Center, Korea Research Institute Bioscience and Biotechnology, Daejeon, Korea;; b Stem Cell Research Center, Korea Research Institute Bioscience and Biotechnology, Daejeon, Korea;; c Institut de Systématique, Evolution, Biodiversité, Muséum national d'Histoire naturelle, UMR 7205 CNRS, Sorbonne Université, EPHE, Paris, France;; d Biodiversity Research Center, Academica Sinica, Taipei, Taiwan

**Keywords:** *Arcoscalpellum epeeum*, barnacle, mitochondrial genome, Scalpellidae, Scalpelliformes

## Abstract

Scalpellids are one of the largest families of Scalpelliformes and reproduce either androdioeciously or dioeciously. Here, we characterized the first mitogenome of a scalpellid barnacle (*Arcoscalpellum epeeum*), which was 15,593 bp in length with a 71.5% AT content. In comparison with the pollicipedids *Capitulum mitella* and *Pollicipes polymerus*, the tRNA genes of *A*. *epeeum* were rearranged between ND3 and ND5, between CYTB and ND1, and between 12S rRNA and ND2. On the mitogenomic tree, the Scalpelliformes families Pollicipedidae and Scalpellidae were not monophyletic, which concurs with previous studies.

Stalked barnacles of the order Scalpelliformes are found in various marine aquatic environments ranging from the intertidal zone to the deep sea and show great reproductive diversity, including hermaphroditism, androdioecy, and dioecy (Lin et al. 2015). Despite revising Scalpelliformes to consist of five families, Calanticidae, Eolepadidae, Lithotryidae, Pollicipedidae, and Scalpellidae, it was still found to be polyphyletic (Buckeridge and Newman 2006; Ahyong et al. 2011; Herrera et al. 2015; Lin et al. 2015). As of 24 August 2018, GenBank contains two complete mitochondrial genomes (mitogenomes) from hermaphrodite pollicipedid barnacles, but no mitogenomes from the other families. Scalpellids are one of the largest families of Scalpelliformes, with over 260 species and have either androdioecious or dioecious reproductive systems (Chan et al. 2014). To understand the phylogenetic relationships and reproductive evolution of Scalpelliformes, we determined the first mitogenome of a deep-sea scalpellid barnacle, *Arcoscalpellum epeeum*, which is androdioecious.

*Arcoscalpellum epeeum* specimens were collected from the Norfolk Ridge between New Caledonia and New Zealand (22°53′S and 167°12′E; 403–429 m depths). The genomic DNA extraction, sequencing, gene annotation, and phylogenetic analyses followed Kim et al. (2017, 2018). The specimen used for the mitogenomic analysis has been deposited in the Biodiversity Research Museum, Academia Sinica, Taiwan (ASIZCR).

The complete mitogenome of *A*. *epeeum* is 15,593 bp in length (71.5% AT content; GenBank accession no. MH791047), consisting of 13 protein-coding genes (PCGs), two ribosomal RNAs (rRNAs), 22 transfer RNAs (tRNAs), and one non-coding region. The intergenic region between tRNA*^Trp^* and ND2 was not determined completely, despite our efforts using Sanger sequencing and comparative mitogenomics.

The gene arrangement and transcriptional polarity showed the ancestral pancrustacean pattern, except for some tRNAs. In comparison with the Scalpelliformes *Capitulum mitella* and *Pollicipes polymerus*, the tRNA genes in the *A*. *epeeum* mitogenome were rearranged between ND3 and ND5, between CYTB and ND1, and between 12S rRNA and ND2. All of the PCGs had an ATN start codon, except COX1, for which the start codon was not determined. Most of the PCGs terminated with a complete stop codon (TAA or TAG), although COX1, COX3, and ND4 had incomplete stop codons (T–). The 16S and 12S rRNAs were 1299 bp (76.3% AT content) and 753 bp (70.8% AT content), respectively. A 118-bp-long (76.3% AT content) non-coding region was located between the 12S rRNA and tRNA*^Ile^*.

A phylogenetic tree was constructed using the PCGs of 18 barnacles using maximum likelihood and Bayesian inference (Figure 1). The scalpellid barnacle *A*. *epeeum* was positioned as an ancestral node of verrucomorph and balanomorph barnacles. Two Scalpelliformes families, Pollicipedidae and Scalpellidae, were not monophyletic, which concurs with previous studies (Pérez-Losada et al. 2008; Linse et al. 2013; Herrera et al. 2015; Lin et al. 2015). In Scalpelliformes, the mitogenomes of Calanticidae, Eolepadidae, and Lithotryidae have not been determined, and further mitogenomic analysis of undetermined taxa is required to deepen our understanding of their phylogeny, sexual evolution, and biogeography.

**Figure 1.**
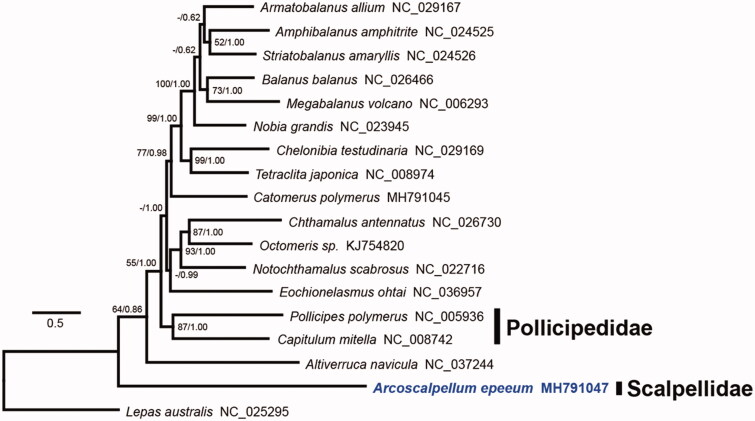
Phylogenetic tree of *Arcoscalpellum epeeum* and other thoracican barnacles based on 13 protein-coding genes from mitogenomes. The model GTR + I + G was selected as the best evolutionary model using jModelTest 2.1.4 (MEGA Inc., Ocheyedan, IA). Numbers at internodes are the maximum-likelihood bootstrap proportions (left) and Bayesian posterior probabilities (right). An asterisk indicates a bootstrap value of less than 50%.

## References

[CIT0001] Ahyong ST, Lowry JK, Alonso M, Bamber RN, Boxshall GA, Castro P, Gerken S, Karaman GS, Goy JW, Jones DS, et al. 2011 Order Scorpiones C.L. Koch, 1850 In: Zhang Z.-Q, editor. Animal biodiversity: an outline of higher-level classification and survey of taxonomic richness. Zootaxa 3148:165–191.

[CIT0002] Buckeridge JS, Newman WA 2006 A revision of the Iblidae and the stalked barnacles (Crustacea: Cirripedia: Thoracica), including new ordinal, familial and generic taxa, and two new species from New Zealand and Tasmanian waters. Zootaxa. 1136:1–38.

[CIT0003] Chan BKK, Corbari L, Rodriguez Moreno PA, Jones DS 2014 Two new deep-sea stalked barnacles, *Arcoscalpellum epeeum* sp. nov. and *Gymnoscalpellum indopacificum* sp. nov. from the Coral Sea, with descriptions of the penis in *Gymnoscalpellum* dwarf males. Zootaxa. 3866:261–276. 25283658 10.11646/zootaxa.3866.2.5

[CIT0004] Herrera S, Watanabe H, Shank TM 2015 Evolutionary and biogeographical patterns of barnacles from deep-sea hydrothermal vents . Mol Ecol. 24:673–689. 25602032 10.1111/mec.13054 PMC5006861

[CIT0005] Kim S-J, Lee W-K, Hou BK, Chan BKK, Ju SJ 2017 Complete mitochondrial genome of the deep-sea asymmetrical barnacle *Altiverruca navicula* (Cirripedia, Thoracica, Verrucumorpha). Mitochondrial DNA B Resour. 2:934–935. 10.1080/23802359.2017.1413297 PMC7800000 33474043

[CIT0006] Kim S-J, Lee W-K, Kim RO, Ju SJ 2018 Complete mitochondrial genome of the hydrothermal vent barnacle *Eochionelasmus ohtai* (Cirripedia, Thoracica). Mitochondrial DNA B Resour. 3:46–47. 10.1080/23802359.2017.1419089 PMC7800951 33474060

[CIT0007] Lin HC, Hoeg JT, Yusa Y, Chan BKK 2015 The origins and evolution of dwarf males and habitat use in thoracican barnacles. Mol Phylogenet Evol. 91:1–11. 25979758 10.1016/j.ympev.2015.04.026

[CIT0008] Linse K, Jackson JA, Fitzcharles E, Sands CJ, Buckeridge JS 2013 Phylogenetic position of Antarctic Scalpelliformes (Crustacea: Cirripedia: Thoracica). Deep Sea Res Part 1 Oceanogr Res Pap. 73:99–116.

[CIT0009] Pérez-Losada M, Harp M, Høeg JT, Achituv Y, Jones D, Watanabe H, Crandall KA 2008 The tempo and mode of barnacle evolution. Mol Phylogenet Evol. 46:328–346. 18032070 10.1016/j.ympev.2007.10.004

